# Comparative Evolution of Duplicated *Ddx3* Genes in Teleosts: Insights from Japanese Flounder, *Paralichthys olivaceus*

**DOI:** 10.1534/g3.115.018911

**Published:** 2015-06-24

**Authors:** Zhongkai Wang, Wei Liu, Huayu Song, Huizhen Wang, Jinxiang Liu, Haitao Zhao, Xinxin Du, Quanqi Zhang

**Affiliations:** Key Laboratory of Marine Genetics and Breeding (MGB), Ministry of Education, College of Marine Life Sciences, Ocean University of China, Qingdao 266003, China

**Keywords:** DDX3, genome duplication, teleost, evolution, subfunctionalization

## Abstract

Following the two rounds of whole-genome duplication that occurred during deuterostome evolution, a third genome duplication event occurred in the stem lineage of ray-finned fishes. This teleost-specific genome duplication is thought to be responsible for the biological diversification of ray-finned fishes. DEAD-box polypeptide 3 (DDX3) belongs to the DEAD-box RNA helicase family. Although their functions in humans have been well studied, limited information is available regarding their function in teleosts. In this study, two teleost *Ddx3* genes were first identified in the transcriptome of Japanese flounder (*Paralichthys olivaceus*). We confirmed that the two genes originated from teleost-specific genome duplication through synteny and phylogenetic analysis. Additionally, comparative analysis of genome structure, molecular evolution rate, and expression pattern of the two genes in Japanese flounder revealed evidence of subfunctionalization of the duplicated *Ddx3* genes in teleosts. Thus, the results of this study reveal novel insights into the evolution of the teleost *Ddx3* genes and constitute important groundwork for further research on this gene family.

Helicase proteins are classified into three superfamilies and two families (SF1−SF5) based on their conserved motifs (Gorbalenya and Koonin 1993), of which motif II and the Walker B motif are found in all helicases (Walker *et al.* 1982). DEAD-box RNA helicases are members of superfamily 2 (SF2) and are characterized by 12 conserved motifs (Linder and Fuller-Pace 2013). The DEAD-box helicase family is named after the amino acid sequence Asp-Glu-Ala-Asp (D-E-A-D) of its motif II (Linder *et al.* 1989). These proteins have important functions in RNA metabolism and are associated with processes involving RNA, such as transcription and degradation (Rocak and Linder 2004). On the basis of their sequences and functions, DEAD-box RNA helicases consist of three subfamilies, including the DEAD-box polypeptide 3 (*Ddx3*) gene family.

The human genome contains two functional genes of the *Ddx3* family: *Ddx3X* located in chromosome X (Park *et al.* 1998) and *Ddx3Y* located in chromosome Y (Lahn and Page 1997). DDX3X is implicated in nucleocytoplasmic shuttling with RNA-dependent ATPase/helicase activity (You *et al.* 1999; Yedavalli *et al.* 2004). DDX3X also reportedly participates in various mRNA and protein biogenesis steps, including transcription (Chao *et al.* 2006), mRNA migration (Kanai *et al.* 2004), pre-mRNA splicing (Zhou *et al.* 2002), and translation (Lai *et al.* 2008), which suggests that DDX3X has a regulatory role in gene expression. In contrast, the human DDX3Y gene, which lies in the azoospermia factor a region, is involved in male fertility. Deletion of *Ddx3Y* results in oligozoospermia, azoospermia, and male sertoli-cell only syndrome (Foresta *et al.* 2001; Kuo *et al.* 2004). Furthermore, the human *Ddx3Y* gene is considered to be one of the genes essential for human spermatogenesis and male fertility. Although several studies have contributed to our understanding of the function of DDX3 in development, studies have been rarely conducted to elucidate the role of the *Ddx3* gene family in teleosts.

Studies have provided evidence of multiple rounds of whole-genome duplication in vertebrate lineages. In particular, two successive rounds of whole-genome duplication are thought to have occurred near the base of vertebrate lineages and played a significant role in vertebrate evolution (Meyer 1998; Hoegg and Meyer 2005; Van De Peer *et al.* 2009; Hoffmann *et al.* 2012). At ∼350Ma, a third-round whole-genome duplication event occurred in the common ancestor of ray-finned fishes (Amores *et al.* 1998; Christoffels *et al.* 2004; Jaillon *et al.* 2004; Vandepoele *et al.* 2004; Meyer and Van De Peer 2005; Brunet *et al.* 2006; Amores *et al.* 2011). Teleost-specific genome duplication (TGD) likely provided gene copies that contributed to evolutionary radiation and phenotypic diversification of teleost fishes. Studies on TGD-derived gene duplicates that evolved distinct physiological or developmental functions in various teleost lineages provide evidence, supporting a cause−effect relationship between gene copy number and species diversity (Braasch *et al.* 2006, 2009; Mulley *et al.* 2006; Hoegg and Meyer 2007; Siegel *et al.* 2007; Liu *et al.* 2009; Liu *et al.* 2013; Li *et al.* 2014). This finding shows that duplicated genes may have diverged from the roles of their ancestors, and this divergence could be demonstrated by changes in evolutionary rates, expression patterns, and regulatory mechanisms. On the basis of a duplication−degeneration−complementation model, duplicated genes may have three main fates, including nonfunctionalization (*i.e.*, one of the two copies is lost or inactivated by mutation), subfunctionalization (*i.e.*, both copies assume more specialized functions than those of their ancestor), and neofunctionalization (*i.e.*, one copy evolves a new function) (Ohno 1970; Hughes 1994; Force *et al.* 1999; Lynch and Conery 2000; Lynch and Force 2000). Studies have revealed a combination of the last two fates, termed subneofunctionalization, when rapid subfunctionalization is accompanied by prolonged and substantial rates of neofunctionalization in duplicated gene evolution (He and Zhang 2005).

In this study, two homologous *Ddx3* genes initially were identified from the transcriptome of Japanese flounder and other teleost genomes. We conducted synteny and phylogenetic analyses of vertebrate *Ddx3* genes. Then, comparisons of genomic structure, molecular evolutionary rate, and expression pattern of the two *Ddx3* genes in Japanese flounder were performed; results revealed probable subfunctionalization of teleost duplicated *Ddx3* genes. This study lays the foundation for evolutionary and functional studies of the *Ddx3* gene family in teleosts.

## Materials and Methods

### Ethics statement

Japanese flounder (*Paralichthys olivaceus*) samples were collected from local aquatic farms. Permission to collect samples was obtained from the local government of Yantai, Shandong, China. All of the procedures complied with and were approved by the Institutional Animal Care and Use Committee of the Ocean University of China.

### Fish

Randomly selected 2-yr-old healthy adult Japanese flounder (five females and five males) were dissected. Muscle, gill, heart, intestine, brain, kidney, liver, spleen, and gonad tissues were collected. Each sample was collected in triplicate. All of the samples were immediately frozen using liquid nitrogen and stored at −80° for total RNA or genomic DNA preparation.

### RNA and genomic DNA extraction

Total RNA was extracted using Trizol Reagent (Invitrogen, Carlsbad, CA) according to the manufacturer’s protocol. The extracted total RNA was treated with RNase-free DNase I (TaKaRa, Dalian, China) to degrade genomic DNA and then frozen at −80°. cDNA was synthesized using 1 µg of total RNA and random hexamer primers with reverse transcriptase M-MLV (RNase H^−^) kit (TaKaRa, Dalian, China) according to the manufacturer’s instructions. Genomic DNA was isolated from muscle tissues by the traditional phenol/chloroform extraction method. The quality and the quantity of RNA and DNA were evaluated by 1.5% agarose gel electrophoresis and spectrophotometry using NanoPhotometer Pearl (Implen, Munich, Germany).

### Data collection

The human *Ddx3X/Y* coding sequences were used as queries for local TBLASTX searches against the transcriptome (SRA, Accession number SRX500343) and genome (unpublished) of Japanese flounder to manually predict putative *Ddx3* genes. Other *Ddx3* genes were identified from TBLASTX search against the genome database at the National Center for Biotechnology Information or Ensembl over the internet. The Assembly ID and Accession numbers are provided in Supporting Information, Table S1.

### Sex-specific amplification of *Ddx3* in Japanese flounder

We designed specific primers (Table S2) based on the genomic sequence of each *Ddx3* gene from Japanese flounder. Polymerase chain reaction (PCR) amplification was performed using the isolated genomic DNA as templates under the following conditions: initial denaturation at 95° for 5 min, followed by 30 cycles at 95° for 30 sec, at 60° for 30 sec, and at 72° for 1 min, and a final extension at 72° for 10 min. PCR products were examined by 1.5% agarose gel electrophoresis.

### Phylogenetic analysis

The coding sequences of vertebrate *Ddx3* genes were aligned in MAFFT v7 (Katoh and Standley 2013). Alignment was carefully checked and gaps and ambiguous sites were removed before phylogenetic analyses were performed. We used ModelGenerator (Keane *et al.* 2006) to choose an appropriate model of sequence evolution for the alignment. The Bayesian method was used as implemented in MrBayes 3.2 (Huelsenbeck *et al.* 2001; Ronquist *et al.* 2012). Maximum likelihood (ML) phylogeny was reconstructed by MEGA6 (Tamura *et al.* 2013), and the branching reliability was tested via bootstrap resampling with 1000 replicates.

### Synteny analysis

Annotated genes surrounding *Ddx3* genes were extracted from the genome databases at the National Center for Biotechnology Information or Ensembl. These genes were mapped according to their relative locations in the chromosome to perform synteny analysis. Then, the Synteny Database was used to detect conserved synteny between chromosomes containing *Ddx3* genes (Catchen *et al.* 2009).

### Genomic structure analysis

The coding sequences of teleost *Ddx3* genes were used as queries for BLASTn searches against the corresponding genomic sequences to find exon−intron boundaries. The genomic structure of each gene was illustrated according to the size and position of exons and introns.

Alignments of the deduced amino acid sequences of teleost DDX3a/b proteins were conducted by MAFFT v7 (Katoh and Standley 2013). Locations of the conserved motifs were marked based on the genomic structure.

### Tests for positive selection of teleost *Ddx3* genes

Alignments, along with Bayesian trees and ML trees, were constructed as described. These alignments were then analyzed with the CODEML package from PAML (Pathology Associated Medical Laboratory) v4.7 (Yang 2007) to estimate the strength and the form of selection among teleost *Ddx3* genes. Nonsynonymous (*d*_N_) and synonymous (*d*_S_) substitution rate ratios (*d*_N_/*d*_S_ or *ω*) were estimated with various site models.

The model M0 assumes a constant *ω* ratio whereas M3 assumes three classes of *ω*. The null model M1a assumes two classes of codon sites for *ω* (0 < ω < 1 and ω = 1), whereas the alternative model M2a assumes one additional class of site with *ω* > 1. The M7 model assumes that *ω* follows a beta distribution with 10 categories, each corresponding to a distinctive *ω* value that is always less than 1, whereas the M8a model allows for an extra class of codons with *ω* = 1. The alternative model M8 has an extra category with *ω* > 1 (Yang *et al.* 2000; Swanson *et al.* 2001; Wong *et al.* 2004). Comparisons between the two nested site models were performed to evaluate the variation in *ω* (M3 *vs.* M0) and to determine the presence of a positively selected class of sites (M2a *vs.* M1a; M8 *vs.* M7 and M8 *vs.* M8a). The M2a−M1a comparison appears to be less powerful than the M8−M7 comparison according to the author (Yang 2007).

Analyses were run starting with branch lengths estimated from the *Ddx3* gene tree and repeated thrice with varying initial starting points of *κ* (transition to transversion rate ratio) and *ω* (0.4, 1, and 4, respectively) as recommended to check multiple local optima. The model pairs were compared using a likelihood ratio test with χ^2^ distribution.

### Tissue distribution pattern of *Ddx3* genes in Japanese flounder

Two specific primer pairs (Table S2) were designed for the Japanese flounder *Ddx3a* and *Ddx3b* genes. A pre-experiment was conducted to confirm single cDNA PCR product and avoid genomic DNA amplification. Specific PCR products were verified by sequencing. Five biological replicates of each sample were analyzed, and each sample was run in triplicate. Quantitative real-time PCR was performed with SYBR Premix Ex *Taq* II (TaKaRa, Dalian, China) by using LightCycler480 (Roche Applied Science, Mannheim, Germany) at 95° (5 min) for preincubation followed by 40 cycles at 95° (15 sec) and 60° (45 sec). The melting curve was analyzed to detect single amplification. Accumulation of fluorescent signal from SYBR Green I was recorded at the 60° (45 sec) phase during each cycle in LightCycler480 Software 1.5. Negative control (no-template reaction) was included.

Relative expression was determined using *18S rRNA* as the reference gene, as previously described (Zhong *et al.* 2008; Gao *et al.* 2013). The target gene was relatively quantified and expressed as fold variation of the reference gene *18S rRNA* by the 2^-ΔΔCq^ comparative Cq method. Data were statistically analyzed by one-way analysis of variance followed by a Tukey’s post-hoc test using SPSS 20.0 (SPSS, Chicago, IL). *P* < 0.05 was considered to indicate statistically significant difference.

## Results

### Identification of *Ddx3* genes

Using TBLASTX searches with E-value at or effectively 0, we identified two *Ddx3* genes from Japanese flounder transcritptome and genome. Two *Ddx3* genes also were identified from the genomes of other teleost species such as zebrafish, torafugu, and Nile tilapia. Spotted gar was the exception, with only a single *Ddx3* gene. In addition to humans, *Ddx3X/Y* could also be found in other eutherians, such as house mouse, cattle, and chimpanzee. Only one *Ddx3* gene was extracted from the genomes of other species, such as elephant shark, coelacanth, and chicken.

Using genomic DNA from male or female Japanese flounder as templates, we found that the amplification products of Japanese flounder *Ddx3* genes showed no sexual specificity (Figure S1).

### Phylogenetic analysis of vertebrate *Ddx3* genes

According to the Akaike Information Criterion and Bayesian Information Criterion from ModelGenerator (Keane *et al.* 2006), both the Bayesian method and the ML method chose the General Time Reversible model and Gamma distributed with Invariant sites (G + I) as the optimal model for *Ddx3* coding sequence alignments.

**Figure 1:**
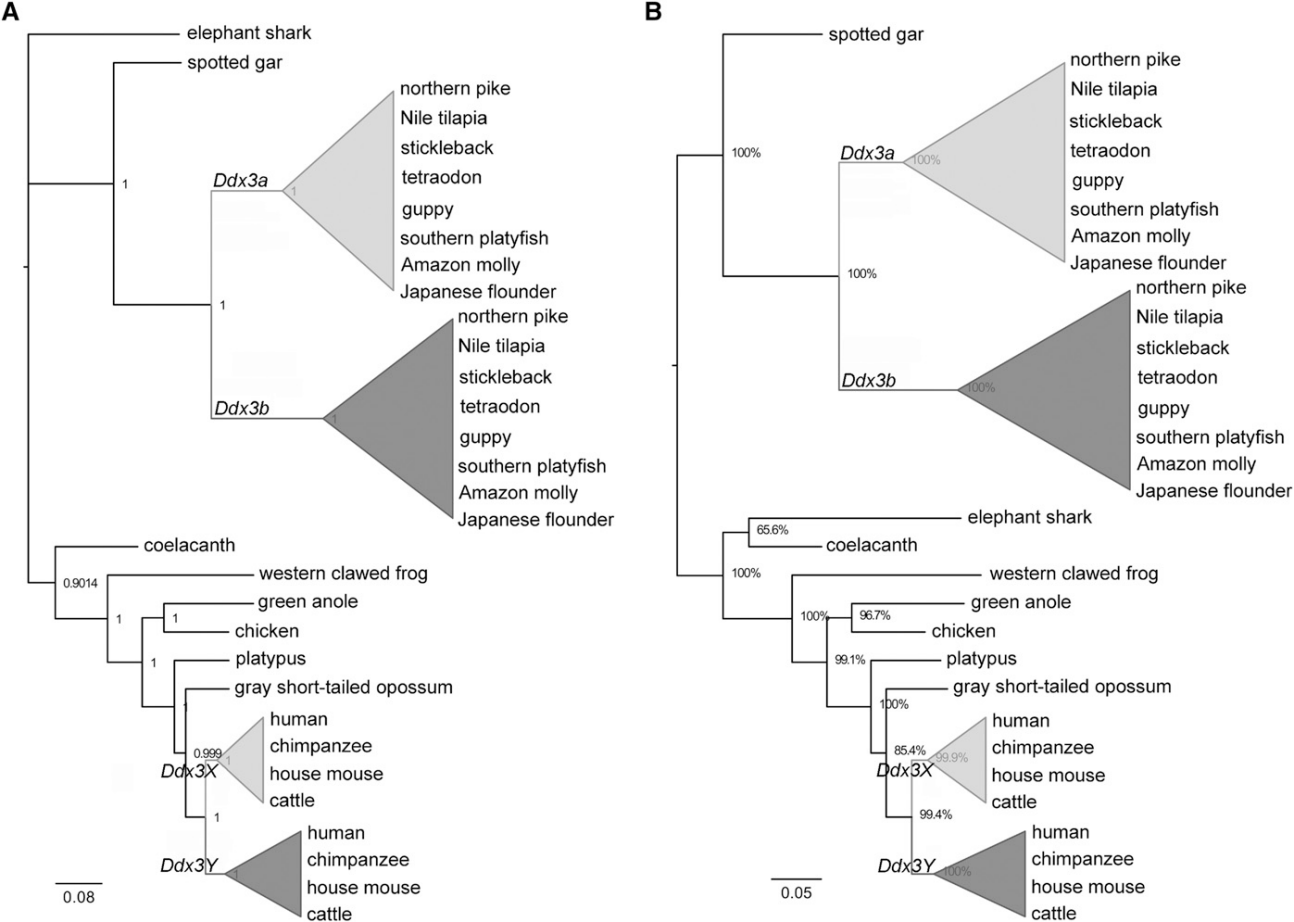
Phylogenetic analyses of vertebrate *Ddx3* genes. (A) Bayesian method was used to construct the gene tree. Numbers at the nodes are Bayesian posterior probabilities. Scale bar = 0.08 substitutions per site. (B) The gene tree was built by the maximum likelihood method. Numbers at the nodes are bootstrap support values with a percentage based on 1000 replicates. Scale bar = 0.05 substitutions per site. Phylogenetic reconstructions were based on the coding sequences of *Ddx3* genes. The accession numbers of these genes at GenBank or Ensembl database are provided in Table S1. Elephant shark *Ddx3* was used as the out-group. Teleost *Ddx3a* or *Ddx3b* genes that clustered together are marked as a cartoon clade, as are the eutherian *Ddx3X/Y* genes.

The result of Bayesian analysis was congruent with accepted species relationships (Figure 1A), whereas the out-group elephant shark was grouped with coelacanth in an analysis based on the ML method (Figure 1B). Nevertheless, both phylogenetic trees were consistent in terms of the topological structure of other species, especially the teleost clade and the eutherian clade. The teleost clade was organized into two clades. The spotted gar *Ddx3* occupied a clade, and the teleost *Ddx3a* genes were clearly separated from *Ddx3b* genes in the other clade. Eutherian *Ddx3* genes were also arranged into two distinct clades: the *Ddx3X* clade and the *Ddx3Y* clade.

### Synteny analyses of *Ddx3* genes

**Figure 2:**
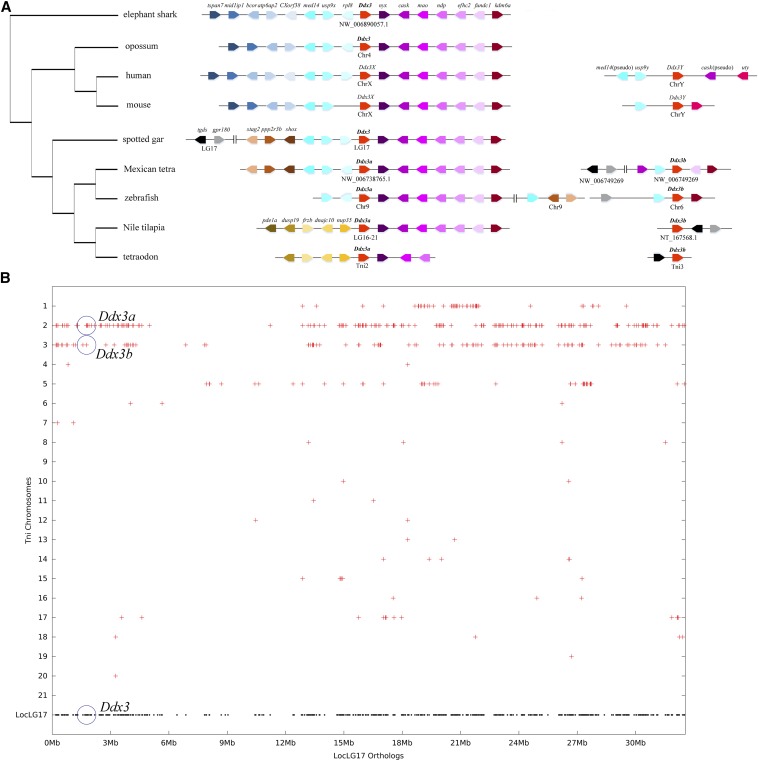
Synteny analyses of vertebrate *Ddx3* genes. (A) Illustration of the syntenic relationship among several *Ddx3* genes, eutherian *DDX3X/Y* genes, and teleost *Ddx3a* and *Ddx3b* genes. The arrows in different colors stand for different genes and the arrowheads point in the direction of the corresponding gene. Gene order was determined according to their relative positions in the same chromosome or scaffold. Vertical lines indicate noncontiguous chromosomal regions. The phylogenetic tree was constructed using the coding sequences of *Ddx3*, *Ddx3a*, and *Ddx3X* from these species by Neighbor Joining method in MEGA6. The Assembly ID at NCBI or Ensembl database was provided in Table S1. (B) Synteny analysis of spotted gar LG17 and tetraodon chromosomes. Spotted gar *Ddx3* gene is at LG17.Tetraodon *Ddx3a* is at Tni2, and *Ddx3b* is at Tni3.

As shown in Figure 2A, the *Ddx3* gene and the surrounding genes were mapped according to their relative locations on the same chromosome or scaffold; vertical lines in the figure indicate noncontiguous chromosomal regions. A conserved syntenic relationship was detected among the *Ddx3* genes from elephant shark, opossum, and *Ddx3X* genes from eutherian. In contrast, the surrounding genes of eutherian *Ddx3Y* changed a lot at the Y chromosome compared with the syntenic genes of *Ddx3X* at the X chromosome. Only the paralogous gene of *usp9x* was retained and others were pseudogenized or deleted.

In teleosts, the downstream genes of *Ddx3* or *Ddx3a* genes also were conserved compared with those in elephant shark, although several downstream genes were lost or shifted in tetraodon (Figure 2A). In contrast, the upstream genes were found to have changed through evolution. From elephant shark to spotted gar, several genes were found to have moved and new genes were found upstream of spotted gar *Ddx3*. The upstream genes of Mexican tetra and zebrafish *Ddx3a* were conserved with those of spotted gar *Ddx3* to some extent, whereas some new genes were translocated upstream of *Ddx3a* in Nile tilapia and tetraodon. Although the syntenic relationship of *Ddx3b* was not consistent among those species, regularities also could be discovered. First, the genes *gpr180* and *tgds* were located in the same chromosome as *Ddx3b* and they often were situated close to *Ddx3b*. Second, paralogous genes of some *Ddx3a* syntenic genes also could be found surrounding *Ddx3b* or away from *Ddx3b* in the same chromosome.

The spotted gar *Ddx3* was located at the chromosome LG17, whereas tetraodon *Ddx3a* and *Ddx3b* were located at chromosomes Tni2 and Tni3, respectively. Using the Synteny Database to detect conserved synteny between spotted gar LG17 and tetraodon chromosomes revealed double conserved synteny among the spotted gar LG17 and the tetraodon chromosomes Tni2 and Tni3 (Figure 2B). In addition, the Tni2 and Tni3 chromosomes also exhibited conserved synteny with each other.

### Genomic structure analyses of teleost *Ddx3* genes

**Figure 3:**
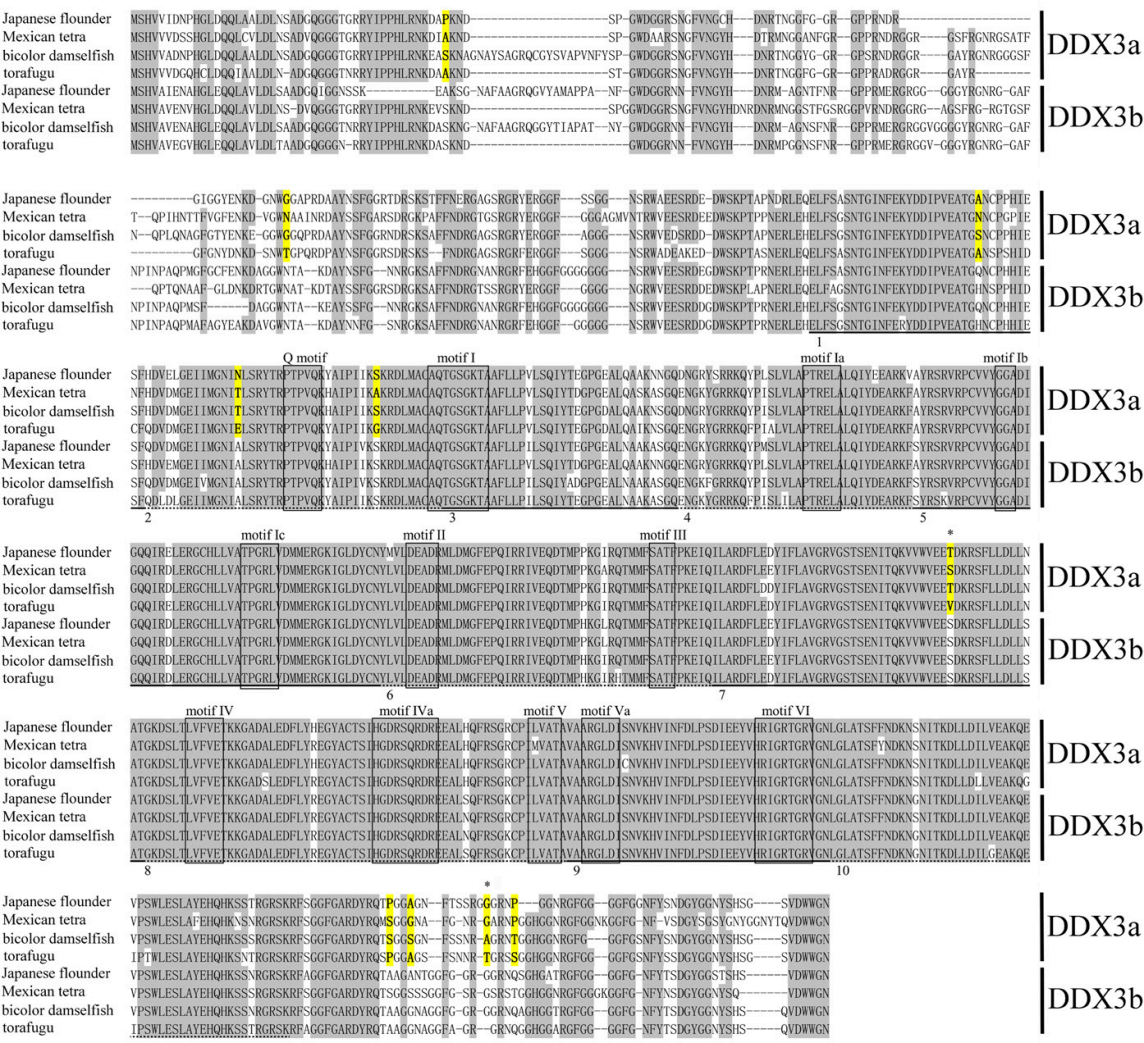
Alignment of the deduced amino acid sequences of teleost *Ddx3* genes. Identical amino acids are in gray background. Amino acid sequences with identical underline are encoded by the same exon. Numbers 1−10 stand for the ten exons of uniform size and position in both *Ddx3a* and *Ddx3b* genes. The 12 conserved motifs characteristic of DEAD-box proteins are boxed. Amino acid sites under positive selection are in yellow background. Asterisk stand for posterior probability > 0.95.

Multiple sequence alignment of deduced full-length DDX3a/b proteins revealed that teleost DDX3a proteins shared higher identity with homologous genes in different species than with DDX3b within the same species (Figure 3). Figure S2 illustrates the genomic structure of *Ddx3* genes in four teleost species. The teleost *Ddx3* genes contained 18−19 exons. In addition, deep analysis of these exons revealed the presence of 10 exons of uniform size and position in both *Ddx3a* and *Ddx3b* genes (Figure 3). Notably, these 10 exons encoded highly identical amino acids containing all the motifs characteristic of DEAD-box proteins (Figure 3).

### Molecular evolutionary analysis of teleost *Ddx3* genes

To explore variation in selective pressure between *Ddx3a* and *Ddx3b* genes in teleosts, we used codon-based models of evolution, as implemented in PAML (Yang 2007). For Bayesian trees of teleost *Ddx3a* and *Ddx3b* genes (Figure S3A and Figure S4A), the results are shown in Table S3. For *Ddx3a* and *Ddx3b*, M0 was rejected in favor of the alternative model M3 (M3 *vs.* M0, *P* < 0.0001), indicating variable selection pressure among sites across these genes. Comparison of M2a *vs.* M1a showed no evidence for positive selection (*P* = 1). For *Ddx3a*, M8 *vs.* M7 and M8 *vs.* M8a comparisons were statistically significant and the model M8 permitting positive selection showed a better fit to the data for *Ddx3a* (*P* < 0.05). For site models, when the likelihood ratio test was significant, the Bayes Empirical Bayes (BEB) tool implemented in PAML (Yang *et al.* 2005) was used to calculate posterior probabilities to identify sites under positive selection (*ω* > 1) in the M2a and M8 models. As a result, 12 sites under positive selection among *Ddx3a* were analyzed by BEB (Table S4). However, neither comparison (M7 *vs.* M8 and M8a *vs.* M8) indicated any site classes as being significantly favored for teleost *Ddx3b* genes (Table S3). This indicated that no amino acid sites were subjected to positive evolution.

For ML trees (Figure S3B and Figure S4B), the results are shown in Table S5. Notably, these results are consistent with the results of Bayesian gene trees. A total of 13 sites in *Ddx3a* genes were found to be under positive selection by BEB (Table S6).

Ten positively selected sites in *Ddx3a* were identified not only from Bayesian tree but also from ML tree; for example, site 429 and site 616 (posterior probability >0.95). All such sites are marked in the amino acid sequence of teleost DDX3a protein (yellow background) in Figure 3. Eight sites including site 616 were at the C- or N-termini, one site was located between motif Q and motif I, and site 429 was located between motif III and motif IV.

### Tissue distribution pattern of Japanese flounder *Ddx3* genes

**Figure 4:**
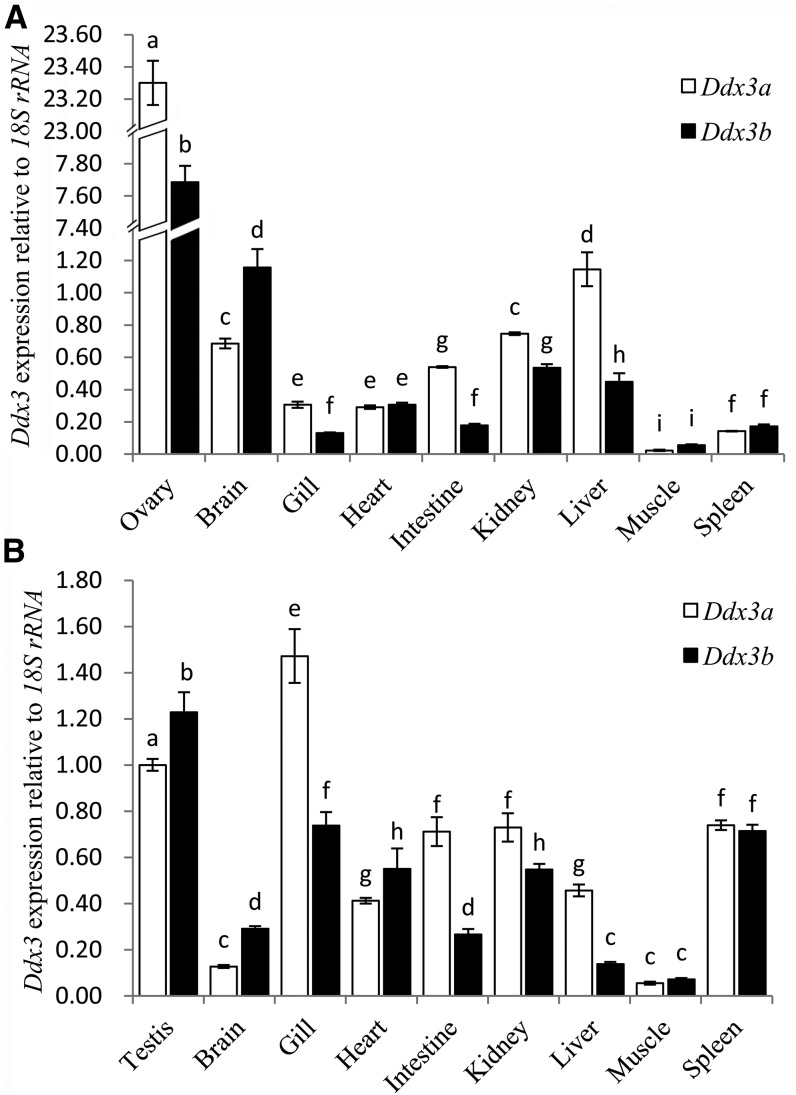
Tissue distribution patterns of Japanese flounder *Ddx3* genes. (A) Relative expression levels of *Ddx3* genes in female tissues. (B) Relative expression levels of *Ddx3* genes in male tissues. Relative expression level of *Ddx3a* in the testis was used as calibrator. Data are shown as mean ± SD (n = 5). Values with different superscripts indicate statistical significance (*P* < 0.05), which was calculated by one-way analysis of variance.

Quantitative real-time PCR results indicated that *Ddx3a* and *Ddx3b* showed differential tissue-specific expression pattern in Japanese flounder. In females (Figure 4A), they both showed very high relative expression levels in the ovary, with the level of *Ddx3a* being much greater than that of *Ddx3b*. In addition, the relative expression level of *Ddx3a* was also greater than that of *Ddx3b* in gills, intestine, kidney, and liver, but lower in the brain. In males (Figure 4B), *Ddx3a* had the greatest relative expression level in gills, whereas *Ddx3b* expression was the greatest in testes. The relative expression level of *Ddx3b* was greater than that of *Ddx3a* in testes and the brain, whereas that of *Ddx3a* was higher in gills, intestine, kidney, and liver.

## Discussion

In this study, Japanese flounder as well as other teleost species were each found to contain two *Ddx3* genes. We therefore thought to explore the evolutionary relationship between teleost *Ddx3a/b* genes.

### Are *Ddx3a*/*b* genes sex-linked in teleosts?

In eutherians, there were also two *Ddx3* genes (*Ddx3X/Y*) found to be separately located on the sex chromosomes. Sexual dimorphism of Japanese flounder has been observed, and its gender is genetically determined on the basis of XX (female)–XY (male) type. However, this fish does not present morphologically differentiated sex chromosomes (Tabata 1991; Mei and Gui 2015). Therefore, we first tested whether the *Ddx3* gene is X- or Y-linked in Japanese flounder. Surprisingly, the PCR-amplification products showed no sexual specificity (Figure S1), demonstrating that neither gene is located on the Y chromosome. Synteny analysis of other teleost *Ddx3a/b* genes also revealed that neither is located in the sex chromosomes (Figure 2A). These results clearly indicated that the teleost *Ddx3a/b* genes are not sex-linked genes as they are in eutherian *Ddx3X/Y*.

### Phylogenetic analysis of teleost *Ddx3a/b* genes

The out-group elephant shark was grouped with coelacanth in our analysis based on the ML method (Figure 1B). This result can be explained by studying the elephant shark genome. Comparison of syntenic genes in elephant shark and their orthologs in human and zebrafish genomes showed that the level of conserved synteny between the elephant shark and humans is higher (Venkatesh *et al.* 2007). Furthermore, in this study, we found a conserved syntenic relationship among the *Ddx3* genes from elephant shark and opossum, as well as *Ddx3X* genes from eutherian. Therefore, it is possible that the elephant shark *Ddx3* gene was clustered with the tetrapod *Ddx3* genes in the phylogenetic analysis. Then, in the phylogenetic analysis based on Bayesian and ML methods, the *Ddx3a* clade was clearly separated from the *Ddx3b* clade under the teleost clade (Figure 1), indicating that *Ddx3a* and *Ddx3b* diverged from a common ancestral gene.

### The origin of teleost *Ddx3a* and *Ddx3b* genes

Eutherians contain another autosomal *Ddx3* gene besides *Ddx3X/Y* (Chang and Liu 2010). This gene is intronless and is thought to derive from retroposition of the *Ddx3X* genes (Mazeyrat *et al.* 1998; Emerson *et al.* 2004; Vong *et al.* 2006). Genomic analysis of the teleost *Ddx3a/b* genes revealed that both contained exon−intron structures (Figure S2). Furthermore, analysis of deduced amino acid sequences suggested that both genes maintained their protein-coding potential (Figure 3), whereas the coding potential of the autosomal *Ddx3* gene in eutherians, except in mice, was diminished (Chang and Liu 2010). On the basis of these results, we conclude that neither of the two teleost *Ddx3* genes evolved from retroposition.

Considering that we could find two *Ddx3* genes only in teleost species that underwent TGD, we hypothesized that the two *Ddx3* genes originated from genome duplication. This could explain why paralogous genes of several *Ddx3a* syntenic genes are located around *Ddx3b* in the same chromosome and why the genes *gpr180* and *tgds* are located near *Ddx3b* despite being far away from the ancestral *Ddx3* gene, as they still are in spotted gar (Figure 2A). For a long period after the chromosome was fully duplicated, recombination must have taken place between the two copies of the duplicated chromosomes and one copy of syntenic genes of *Ddx3a* and *Ddx3b* may have been pseudogenized, deleted, or translocated. It is possible that the chromosome corresponding to the *Ddx3a* gene retained more of the ancestral genes, although the extent of this is clearly lineage-dependent. Similarly, one copy each of *gpr180* and *tgds* appear to have been lost, and the other copies of *gpr180* and *tgds* were likely translocated closer to post-duplication *Ddx3b*.

Only one *Ddx3* gene is located in chromosome LG17 of spotted gar, which did not undergo TGD, whereas tetraodon *Ddx3a* is located in chromosome Tni2 and *Ddx3b* is located in chromosome Tni3. Synteny analysis revealed that the tetraodon chromosomes Tni2 and Tni3 share conserved synteny with each other and with the spotted gar LG17 (Figure 2B). It is noteworthy here that Tni2 and Tni3 were previously shown to be derived from the ancestral chromosome *c* and duplicated during TGD (Kasahara *et al.* 2007). Therefore, our evidence showing conserved synteny between these chromosomes further confirms that the ancestral *Ddx3* gene was duplicated during TGD. Our result also rules out the possibility that *Ddx3a/b* just originated from large-scale segmental duplication through evolution.

To further confirm these findings, dot plots of the spotted gar LG17 with stickleback were performed. Our results showed another double conserved synteny between GacgroupI and GacgroupXVI (Figure S5A). *Ddx3a* is found in groupXVI in stickleback, and strong synteny was observed between Tni2 and GacgroupXVI (Figure S5B). *Ddx3b* is found in groupI in stickleback and unequivocal evidence of synteny between Tni3 and GacgroupI was observed (Figure S5C). These results present strong evidence of whole-genome duplication that occurred specifically in teleosts.

### Subfunctionalization of the duplicated teleost *Ddx3* genes

The 10 uniform exons of *Ddx3* genes encode highly homologous amino acids that span the entire conserved motifs and domains characteristic of DEAD-box protein (Figure 3), suggesting conserved genomic structure and protein function between teleost *Ddx3a* genes and *Ddx3b* genes.

No models of selection were statistically significant for the teleost *Ddx3b* genes (Table S3 and Table S5), suggesting probable purifying selection of *Ddx3b* during evolution. In contrast, 10 positively selected sites (Figure 3) can be identified in DDX3a, suggesting divergent evolutionary fates between *Ddx3a* and *Ddx3b*. In addition, the C- or N-termini of DEAD box helicases can facilitate RNA binding (Mohr *et al.* 2008). The Q motif and motif I regulate ATP binding and hydrolysis (Tanner *et al.* 2003; Cordin *et al.* 2004). The linker between motif III and motif IV connects the two core domains of DEAD box helicase and can regulate their orientation (Andreou and Klostermeier 2012). Therefore, mutation of these positively selected sites may have affected the RNA helicase and ATPase activities of DDX3a, resulting in functional divergence between DDX3a and DDX3b.

The function of DDX3 proteins in gametogenesis has been well described in other metazoans (Leroy *et al.* 1989; Mazeyrat *et al.* 1998; Mochizuki *et al.* 2001; Session *et al.* 2001; Johnstone *et al.* 2005). In this study, we found differential tissue-specific expression patterns of *Ddx3a* and *Ddx3b*, especially in the gonads, in Japanese flounder (Figure 4). The relative expression level of *Ddx3a* was much greater than that of *Ddx3b* in the ovary, whereas that of *Ddx3b* was greater than that of *Ddx3a* in the testis. These results provide evidence for divergent functions between DDX3a and DDX3b in teleost gametogenesis. However, this remains to be directly addressed through *in vitro* and *in vivo* analysis of the exact functions of DDX3a and DDX3b in suitable fish models.

Considering the aforementioned results, we conclude that the teleost DDX3a and DDX3b proteins retained the conserved function of DEAD-box RNA helicases, whereas their divergent evolutionary fates resulted in their functional differences. Thus, there is sufficient evidence for subfunctionalization of the duplicated *Ddx3* genes in teleosts after TGD.

In summary, we investigated the origin of teleost *Ddx3a/b* genes in the current study. It is the first to report that two *Ddx3* genes are present in teleosts as a result of TGD. In addition, our findings suggest probable subfunctionalization of the duplicated *Ddx3* genes in teleost through evolution. Therefore, this study provides novel insights into the teleost *Ddx3* gene family and open doors to further functional studies in suitable fish models.

## Supplementary Material

Supporting Information
