# Supplementary material for: Comparative Evolution of Duplicated Ddx3 Genes in Teleosts: Insights from Japanese Flounder, Paralichthys olivaceus
Source: G3 (Bethesda). 2015 Jun 24;5(8):1765–73. doi: 10.1534/g3.115.018911 (PMC4528332; doi:10.1534/g3.115.018911)
Supplement: Supporting Information [file supp_5_8_1765__index.html]

Comparative Evolution of Duplicated Ddx3 Genes in Teleosts: Insights from Japanese Flounder, Paralichthys olivaceus — Supporting Information 

# Comparative Evolution of Duplicated *Ddx3* Genes in Teleosts: Insights from Japanese Flounder, *Paralichthys olivaceus*

## Supporting Information for Wang *et al.*, 2015

**Files in this Data Supplement:**

- Supporting Information - Figures S1-S5 and Tables S1-S6 (PDF, 687 KB)
- Figure S1 - Sex-specific amplification of *Ddx3* genes in Japanese flounder. (PDF, 337 KB)
- Figure S2 - Genomic structure of teleost *Ddx3a* and *Ddx3b* genes. (PDF, 212 KB)
- Figure S3 - Phylogenetic tree of teleost *Ddx3a* genes used in PAML analysis. (PDF, 177 KB)
- Figure S4 - Phylogenetic tree of teleost *Ddx3b* genes used in PAML analysis. (PDF, 177 KB)
- Figure S5 - Detection of conserved chromosome among teleosts. (PDF, 270 KB)
- Table S1 - List of taxa used in this study. (PDF, 156 KB)
- Table S2 - Sequences of primers used for cloning Japanese flounder *Ddx3* genes. (PDF, 150 KB)
- Table S3 - Results of site model analyses on teleost *Ddx3* Bayesian gene trees. (PDF, 158 KB)
- Table S4 - Sites selected in *Ddx3a* by BEB in Bayesian tree. (PDF, 177 KB)
- Table S5 - Results of site model analyses on teleost *Ddx3* ML gene trees. (PDF, 158 KB)
- Table S6 - Sites selected in *Ddx3a* by BEB in ML tree. (PDF, 149 KB)
