# Supplementary material for: Comparative Evolution of Duplicated Ddx3 Genes in Teleosts: Insights from Japanese Flounder, Paralichthys olivaceus
Source: G3 (Bethesda). 2015 Jun 24;5(8):1765–73. doi: 10.1534/g3.115.018911 (PMC4528332; doi:10.1534/g3.115.018911)
Supplement: Supporting Information [file supp_g3.115.018911_018911SI.pdf]

G3: Genes | Genomes | Genetics

Comparative Evolution of Duplicated *Ddx3* Genes in Teleosts: Insights from Japanese flounder, *Paralichthys olivaceus*

Zhongkai Wang, Wei Liu, Huayu Song, Huizhen Wang, Jinxiang Liu, Haitao Zhao, Xinxin Du, Quanqi Zhang

Key Laboratory of Marine Genetics and Breeding (MGB), Ministry of Education, College of Marine Life Sciences, Ocean University of China, Qingdao 266003, China

Corresponding author: Quanqi Zhang

Ocean University of China, No 5 Yushan Road, Qingdao 266003, China.

Tel./fax: +86 532 82031806

E-mail: qzhang@ouc.edu.cn

DOI: 10.1534/g3.115.018911

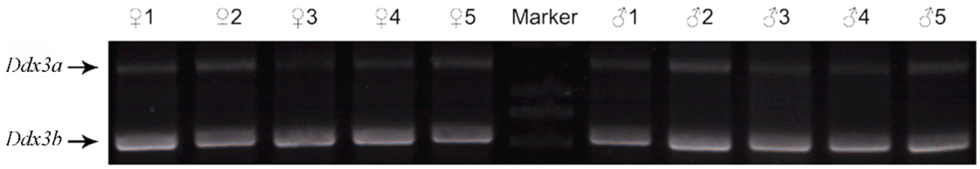

Figure S1 Sex-specific amplification of *Ddx3* genes in Japanese flounder. Using the genomic DNA of females or males as templates, the amplification products of *Ddx3* genes showed no sexual specificity.

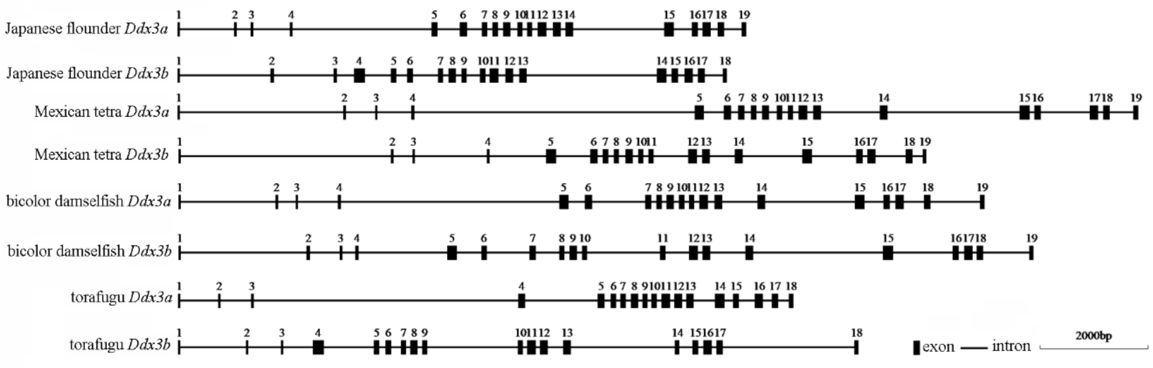

Figure S2 Genomic structure of teleost *Ddx3a* and *Ddx3b* genes.

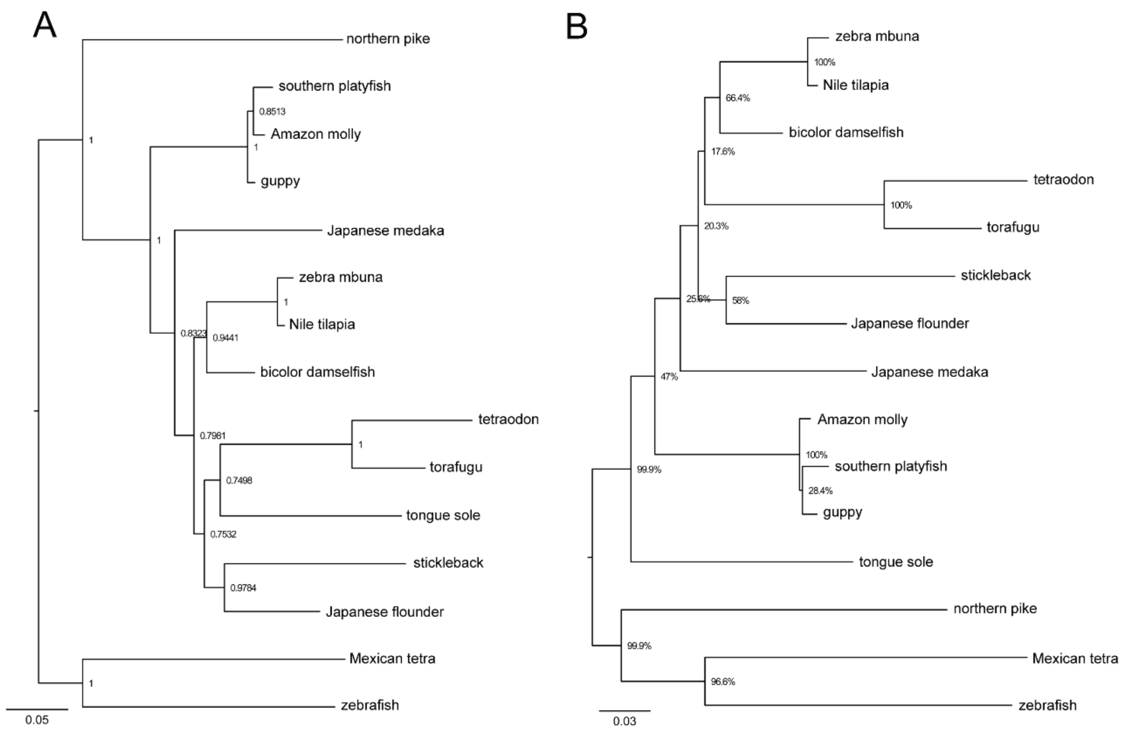

Figure S3 Phylogenetic tree of teleost *Ddx3a* genes used in PAML analysis. (A) Bayesian tree of teleost *Ddx3a* genes. Numbers at the nodes are Bayesian posterior probabilities. Scale bar = 0.05. (B) Maximum likelihood tree of teleost *Ddx3a* genes. Numbers at the nodes are bootstrap support values. Scale bar = 0.03. Phylogenetic reconstructions were based on the coding sequences of *Ddx3a* genes. The accession numbers of these genes at GenBank or Ensembl database are provided in Table S1.

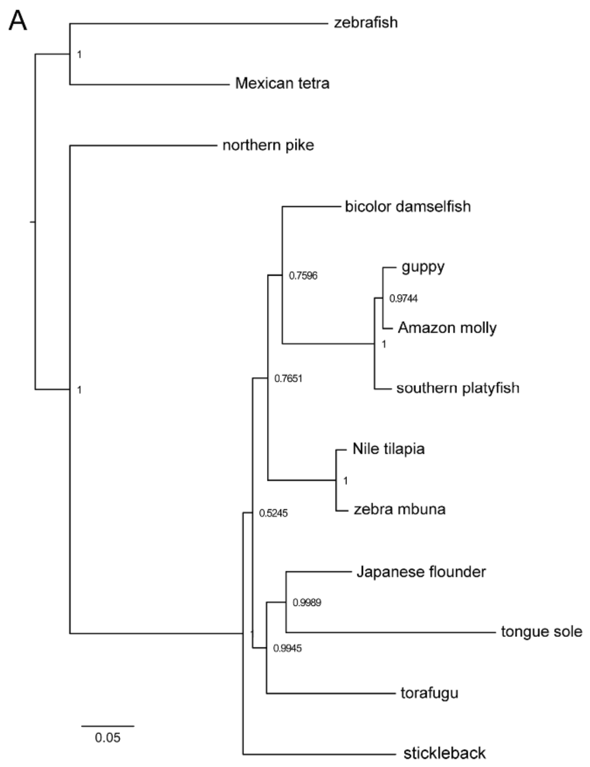

Figure S4 Phylogenetic tree of teleost *Ddx3b* genes used in PAML analysis. (A) Bayesian tree of teleost *Ddx3b* genes. Numbers at the nodes are Bayesian posterior probabilities. Scale bar = 0.05. (B) Maximum likelihood tree of teleost *Ddx3b* genes. Numbers at the nodes are bootstrap support values. Scale bar = 0.04. Phylogenetic reconstructions were based on the coding sequences of *Ddx3b* genes. The accession numbers of these genes at GenBank or Ensembl database are provided in Table S1.

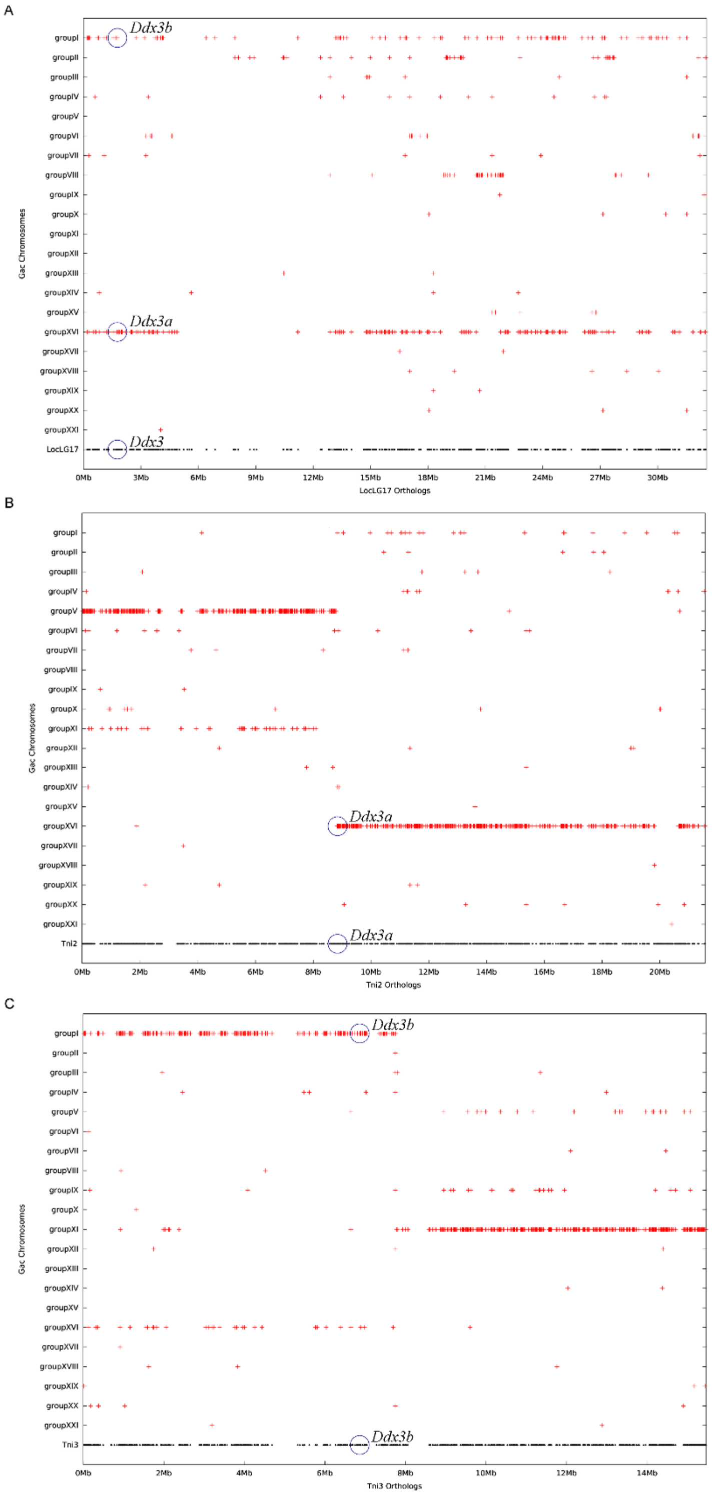

Figure S5 Detection of conserved chromosome among teleosts. Synteny analysis of spotted gar LG17 (A), tetraodon Tni2 (B), and tetraodon Tni3 (C) and stickleback chromosomes. Stickleback *Ddx3a* is in groupXVI and *Ddx3b* is in groupI.

Table S1 List of taxa used in this study.

Organism Name	Common Name	Assembly ID	Gene	Accession No.
Homo sapiens	human	GRCh38	*Ddx3X*	NM_001356.3
			Ddx3Y	*NM_001122665.1*
Mus musculus	*house mouse*	*GRCm38.p2*	*Ddx3X*	*XM_006527566.1*
			Ddx3Y	XM_006531605.1
Bos taurus	cattle	Bos_taurus_UMD_3.1.1	*Ddx3X*	XM_005228286.2
			Ddx3Y	XM_005228498.2
Pan troglodytes	chimpanzee	Pan_troglodytes-2.1.4	*Ddx3X*	XM_009438962.1
			Ddx3Y	XM_009439896.1
Monodelphis domestica	gray short-tailed opossum	MonDom5	*Ddx3*	XM_007493339.1
Ornithorhynchus anatinus	platypus	Ornithorhynchus_ anatinus-5.0.1	*Ddx3*	XM_007667286.1
Gallus gallus	chicken	Gallus_gallus-4.0	*Ddx3*	NM_001030800.1
Anilis carolinesis	green anole	AnoCar2.0	*Ddx3*	XM_008107404.1
Xenopus tropicalis	western clawed frog	Xtropicalis_v7	*Ddx3*	NM_203865.1
Latimeria chalumnae	coelacanth	LatCha1	*Ddx3*	XM_006005839.1
Callorhinchus milii	elephant shark	Callorhinchus_ milii-6.1.3	*Ddx3*	XM_007901579.1
Lepisosteus oculatus	spotted gar	LepOcu1	*Ddx3*	XM_006638889.1
Danio rerio	zebrafish	GRCz10	*Ddx3a*	NM_130941.2
			Ddx3b	NM_001126423.1
Astyanax mexicanus	Mexican tetra	Astyanax_	*Ddx3a*	XM_007227851.1
		mexicanus-1.0.2	*Ddx3b*	XM_007244224.1
Esox lucius	northern pike	EsoLuc1.0	*Ddx3a*	XM_010889977.1
			Ddx3b	XM_010886335.1
Stegastes partitus	bicolor damselfish	Stegastes_partitus-1.0.2	*Ddx3a*	XM_008279959.1
			Ddx3b	XM_008292391.1
Poecilia reticulate	guppy	Guppy_female_1.0+MT	*Ddx3a*	XM_008437108.1

			Ddx3b	XM_008426133.1
Oreochromis niloticus	Nile tilapia	Orenil1.1	*Ddx3a*	XM_005475255.1
			Ddx3b	NT_167568.1
Cynoglossus semilaevis	tongue sole	Cse_v1.0	*Ddx3a*	XM_008327813.1
			Ddx3b	XM_008324681.1
Takifugu rubripes	torafugu	FUGU5	*Ddx3a*	XM_003961572.1
			Ddx3b	XM_003966411.1
Oryzias latipes	Japanese medaka	ASM31367v1	*Ddx3a*	XM_004081602.1
			Ddx3b	XM_004085517.1
Paralichthys olivaceus	Japanese flounder	unpublished	*Ddx3a*	KP205082
			Ddx3b	KP205083
Poecilia formosa	Amazon molly	Poecilia_formosa-5.1.2	*Ddx3a*	XM_007571428.1
			Ddx3b	XM_007572225.1
Xiphophorus maculatus	southern platyfish	Xiphophorus_	*Ddx3a*	XM_005803788.1
		maculatus-4.4.2	*Ddx3b*	XM_005813094.1
Maylandia zebra	zebra mbuna	MetZeb1.1	*Ddx3a*	XM_004566279.1
			Ddx3b	XM_004573843.1
Tetraodon nigroviridis	tetraodon	TETRAODON 8.0	*Ddx3a*	ENSTNIG00000011569
			Ddx3b	ENSTNIG00000014514
Gasterosteus aculeatus	stickleback	BROAD S1	*Ddx3a*	ENSGACG00000008206
			Ddx3b	ENSGACG00000014407

Table S2 Sequences of primers used for cloning Japanese flounder *Ddx3* genes.

Primers	Sequence(5'-3')	Product	usage
ddx3-1_FW	TTTCTGTACCACGAAGGTTACGC	1370 bp	PCR
ddx3-1_RV	CATAGCCGTCATTGCTGTAGAAG		
ddx3-2_FW	ATTGGAACAGGAGCATTTGACC	512 bp	PCR
ddx3-2_RV	GTCAGAGGCTTTGAATGGGGT		
DDX3-1-FW	GATGCTCCAAAAAACGATTCAC	128 bp	RT-qPCR
DDX3-1-RV	ATTCCTCTATCATTGCGAGGG		
DDX3-2-FW	ATGCCATTCCAATCGTCAAG	289 bp	RT-qPCR
DDX3-2-RV	TACACAACACAGGGACGCACT		

Table S3 Results of site model analyses on teleost *Ddx3* Bayesian gene trees.

Tree	Model	ln*L*	*κ*	Parameters[a]			Null	LRT	df	*P*
				ω_0/p	ω_1/q	ω_2/ω_p				
Ddx3a	M0	-12032.610	1.827	0.077			n/a			
	M1a	-11821.569	1.941	0.042(89.94%)	1.000(10.06%)		n/a			
	M2a	-11821.569	1.941	0.042(89.94%)	1.000(10.06%)	87.302(0)	M1a	0	2	**1.000**
	M3	-11730.857	1.829	0.013(74.72%)	0.224(20.60%)	0.750(4.68%)	M0	603.506	4	**0**
	M7	-11734.989	1.823	0.198	1.828		n/a			
	M8a	-11739.484	1.849	0.223	2.257	1.000(3.71%)	n/a			
	M8	-11731.240	1.825	0.207	2.044	2.600(0.35%)	M7	7.498	2	**0.024**
							M8a	16.488	1	**0**
Ddx3	M0	-10420.556	1.782	0.057			n/a			
	M1a	-10373.0	1.857	0.046(95.77%)	1.000(4.23%)		n/a			

	22								
M2a	-10373.022	1.857	0.046(95.77%)	1.000(4.23%)	48.098 (0)	M1a	0	2	**1.000**
M3	-10288.100	1.775	0(52.95%)	0.084(37.85%)	0.324(9.20%)	M0	264.912	4	**0**
M7	-10288.873	1.776	0.286	4. 080		n/a			
M8a	-10287.692	1.774	0.230	2. 738	1.000(0.65%)	n/a			
M8	-10286.625	1.782	0.306	4.687	1.449(0.41%)	M7	4.496	2	**0.106**
						M8a	2.134	1	**0.144**

Abbreviations: **ln*L***, *ln* likelihood; ***κ***, transition/transversion ratio; **LRT**, likelihood ratio test statistic; **df**, degrees of freedom; ***P***, P-value; **n/a**, not applicable.

[a] : ω values of each site class are shown for models M0-M3 (ω_0-ω_2) with the proportion of each site class in parentheses. For M7-M8, the shape parameters, p and q, which describe the beta distribution are listed. In addition, the ω value for the positively selected site class (ω_p, with the proportion of sites in parentheses) is shown for M8a (where ω_p is constrained to equal one) and M8.

Table S4 Sites selected in *Ddx3a* by BEB in Bayesian tree.

Model	codon site	BEB		
		Pr(ω>1)	post mean	+/-
M8	43	0.513	1.070	0.459
	89	0.583	1.132	0.451
	131	0.578	1.135	0.443
	151	0.577	1.140	0.436
	176	0.860	1.392	0.275
	199	0.571	1.136	0.435
	219	0.562	1.124	0.441
	429	0.962*	1.473	0.147
	607	0.539	1.093	0.458
	609	0.560	1.089	0.483
	616	0.980*	1.486	0.109
	620	0.763	1.306	0.358

*: posterior probability > 0.95

Table S5 Results of site model analyses on teleost *Ddx3* ML gene trees.

Tree	Model	ln*L*	*κ*	Parameters[a]			Null	LRT	df	*P*
				ω_0/p	ω_1/q	ω_2/ω_p				
Ddx3a	M0	-12069.904	1.870	0.079			n/a			
	M1a	-11828.656	1.967	0.042(90.37%)	1.000(9.63%)		n/a			
	M2a	-11828.656	1.967	0.042(90.37%)	1.000(9.63%)	37.503(0)	M1a	0	2	1.000
	M3	-11746.341	1.872	0.013(74.49%)	0.217(20.62%)	0.813(4.89%)	M0	647.126	4	0
	M7	-11753.109	1.865	0.190	1.672		n/a			
	M8a	-11753.656	1.891	0.224	2.438	1.000(4.47%)	n/a			
	M8	-11745.686	1.873	0.242	3.063	1.000(2.94%)	M7	14.846	2	0
							M8a	15.940	1	0
Ddx3b	M0	-10427.457	1.799	0.057			n/a			
	M1a	-10379.321	1.873	0.046(95.89%)	1.000(4.11%)		n/a			
	M2a	-10379.321	1.873	0.046(95.89%)	1.000(4.11%)	50.201 (0)	M1a	0	2	1.000
	M3	-10291.089	1.797	0.010(71.32%)	0.178(28.17%)	1.431(0.51%)	M0	272.736	4	0
	M7	-10293.577	1.793	0.285	4. 030		n/a			
	M8a	-10292.146	1.791	0.230	2. 714	1.000(0.67%)	n/a			
	M8	-	1.800	0.306	4.651	1.494(0.42%)	M7	4.892	2	0.087

10291.131				
	M8a	2.03	1	**0.154**

Abbreviations: **ln*L***, *ln* likelihood; ***κ***, transition/transversion ratio; **LRT**, likelihood ratio test statistic; **df**, degrees of freedom; ***P***, P-value; **n/a**, not applicable.

[a] : ω values of each site class are shown for models M0-M3 (ω_0-ω_2) with the proportion of each site class in parentheses. For M7-M8, the shape parameters, p and q, which describe the beta distribution are listed. In addition, the ω value for the positively selected site class (ω_p, with the proportion of sites in parentheses) is shown for M8a (where ω_p is constrained to equal one) and M8.

Table S6 Sites selected in *Ddx3a* by BEB in ML tree.

Model	codon site	BEB		
		Pr(ω>1)	post mean	+/-
M8	9	0.585	1.127	0.460
	43	0.604	1.157	0.438
	89	0.581	1.129	0.453
	111	0.651	1.198	0.426
	123	0.842	1.368	0.314
	176	0.850	1.383	0.285
	199	0.765	1.309	0.353
	219	0.654	1.211	0.409
	429	0.983*	1.488	0.094
	607	0.791	1.329	0.341
	609	0.544	1.071	0.488
	616	0.984*	1.489	0.092
	620	0.837	1.370	0.301

*: posterior probability > 0.95
