# Supplementary material for: Comparative Evolution of Duplicated Ddx3 Genes in Teleosts: Insights from Japanese Flounder, Paralichthys olivaceus
Source: G3 (Bethesda). 2015 Jun 24;5(8):1765–73. doi: 10.1534/g3.115.018911 (PMC4528332; doi:10.1534/g3.115.018911)
Supplement: Supporting Information [file supp_g3.115.018911_FigureS1.pdf]

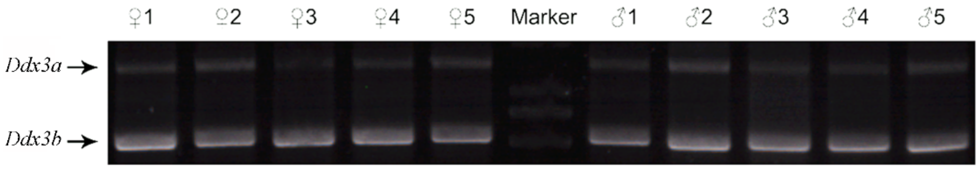

Figure S1 Sex-specific amplification of *Ddx3* genes in Japanese flounder. Using the genomic DNA of females or males as templates, the amplification products of *Ddx3* genes showed no sexual specificity.
