# Supplementary figures and images for: Comparative Evolution of Duplicated Ddx3 Genes in Teleosts: Insights from Japanese Flounder, Paralichthys olivaceus
Source: G3 (Bethesda). 2015 Jun 24;5(8):1765–73. doi: 10.1534/g3.115.018911 (PMC4528332; doi:10.1534/g3.115.018911)

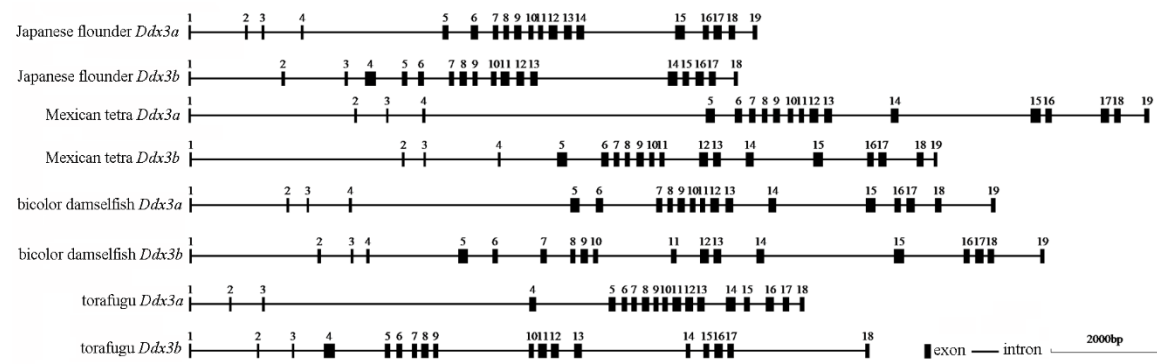

**Figure S2** Genomic structure of teleost *Ddx3a* and *Ddx3b* genes.

Supplement: Supporting Information [file supp_g3.115.018911_FigureS2.pdf]
