# Supplementary material for: Comparative Evolution of Duplicated Ddx3 Genes in Teleosts: Insights from Japanese Flounder, Paralichthys olivaceus
Source: G3 (Bethesda). 2015 Jun 24;5(8):1765–73. doi: 10.1534/g3.115.018911 (PMC4528332; doi:10.1534/g3.115.018911)
Supplement: Supporting Information [file supp_g3.115.018911_FigureS3.pdf]

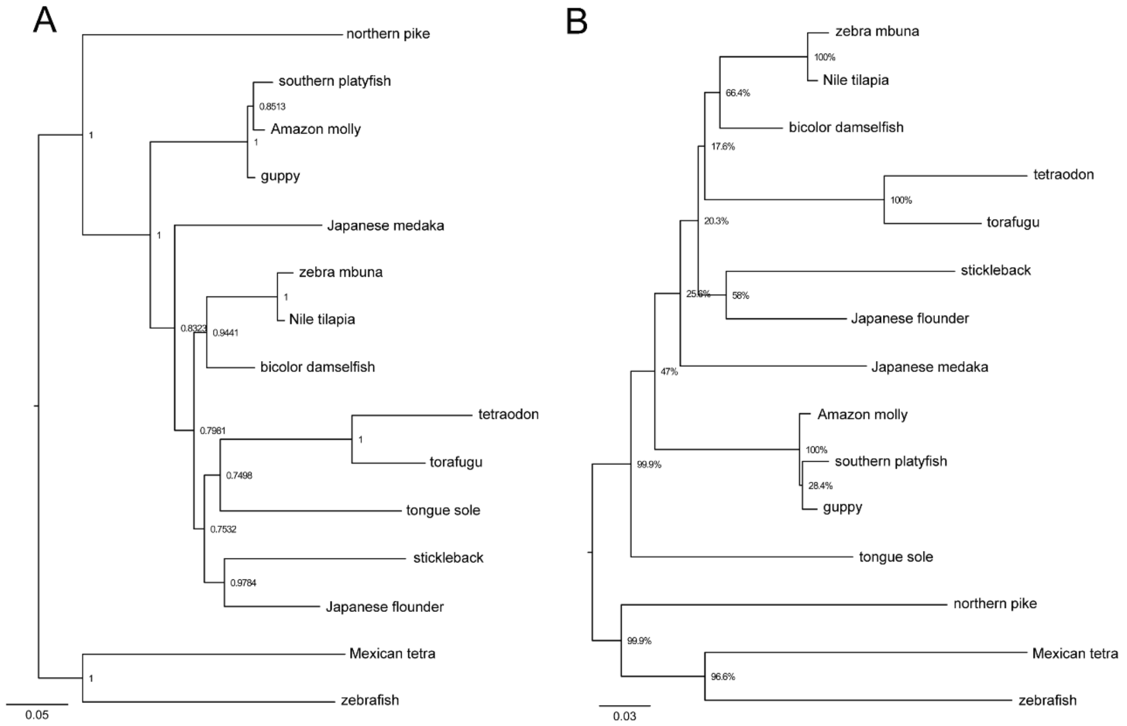

Figure S3 Phylogenetic tree of teleost *Ddx3a* genes used in PAML analysis. (A) Bayesian tree of teleost *Ddx3a* genes. Numbers at the nodes are Bayesian posterior probabilities. Scale bar = 0.05. (B) Maximum likelihood tree of teleost *Ddx3a* genes. Numbers at the nodes are bootstrap support values. Scale bar = 0.03. Phylogenetic reconstructions were based on the coding sequences of *Ddx3a* genes. The accession numbers of these genes at GenBank or Ensembl database are provided in Table S1.
