# Supplementary material for: Comparative Evolution of Duplicated Ddx3 Genes in Teleosts: Insights from Japanese Flounder, Paralichthys olivaceus
Source: G3 (Bethesda). 2015 Jun 24;5(8):1765–73. doi: 10.1534/g3.115.018911 (PMC4528332; doi:10.1534/g3.115.018911)
Supplement: Supporting Information [file supp_g3.115.018911_FigureS5.pdf]

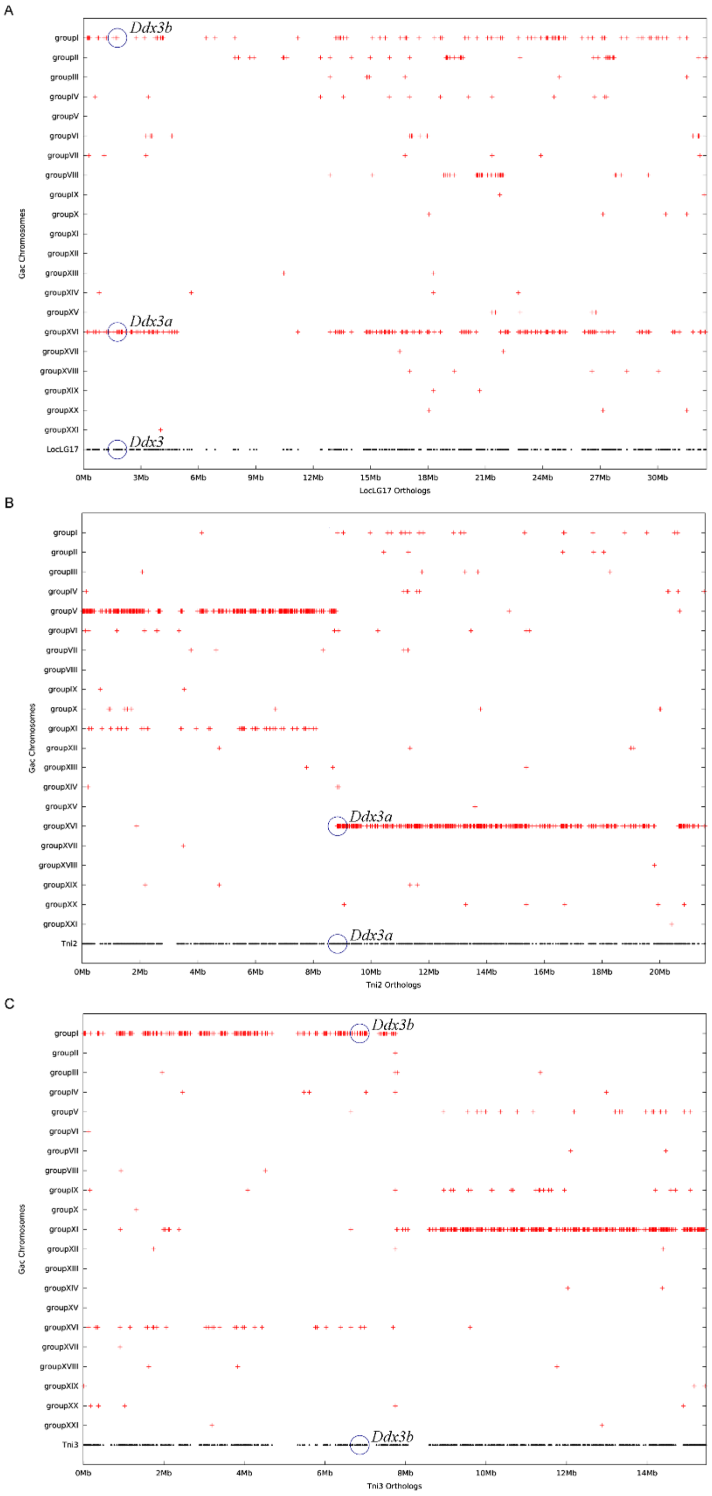

Figure S5 Detection of conserved chromosome among teleosts. Synteny analysis of spotted gar LG17 (A), tetraodon Tni2 (B), and tetraodon Tni3 (C) and stickleback chromosomes. Stickleback *Ddx3a* is in groupXVI and *Ddx3b* is in groupI.
