# Supplementary material for: Comparative Evolution of Duplicated Ddx3 Genes in Teleosts: Insights from Japanese Flounder, Paralichthys olivaceus
Source: G3 (Bethesda). 2015 Jun 24;5(8):1765–73. doi: 10.1534/g3.115.018911 (PMC4528332; doi:10.1534/g3.115.018911)
Supplement: Supporting Information [file supp_g3.115.018911_TableS1.pdf]

Table S1 List of taxa used in this study.

Organism Name	Common Name	Assembly ID	Gene	Accession No.
Homo sapiens	human	GRCh38	*Ddx3X*	NM_001356.3
			Ddx3Y	*NM_001122665.1*
Mus musculus	*house mouse*	*GRCm38.p2*	*Ddx3X*	*XM_006527566.1*
			Ddx3Y	XM_006531605.1
Bos taurus	cattle	Bos_taurus_UMD_3.1.1	*Ddx3X*	XM_005228286.2
			Ddx3Y	XM_005228498.2
Pan troglodytes	chimpanzee	Pan_troglodytes-2.1.4	*Ddx3X*	XM_009438962.1
			Ddx3Y	XM_009439896.1
Monodelphis domestica	gray short-tailed opossum	MonDom5	*Ddx3*	XM_007493339.1
Ornithorhynchus anatinus	platypus	Ornithorhynchus_ anatinus-5.0.1	*Ddx3*	XM_007667286.1
Gallus gallus	chicken	Gallus_gallus-4.0	*Ddx3*	NM_001030800.1
Anilis carolinesis	green anole	AnoCar2.0	*Ddx3*	XM_008107404.1
Xenopus tropicalis	western clawed frog	Xtropicalis_v7	*Ddx3*	NM_203865.1
Latimeria chalumnae	coelacanth	LatCha1	*Ddx3*	XM_006005839.1
Callorhinchus milii	elephant shark	Callorhinchus_ milii-6.1.3	*Ddx3*	XM_007901579.1
Lepisosteus oculatus	spotted gar	LepOcu1	*Ddx3*	XM_006638889.1
Danio rerio	zebrafish	GRCz10	*Ddx3a*	NM_130941.2
			Ddx3b	NM_001126423.1
Astyanax mexicanus	Mexican tetra	Astyanax_	*Ddx3a*	XM_007227851.1
		mexicanus-1.0.2	*Ddx3b*	XM_007244224.1
Esox lucius	northern pike	EsoLuc1.0	*Ddx3a*	XM_010889977.1
			Ddx3b	XM_010886335.1
Stegastes partitus	bicolor damselfish	Stegastes_partitus-1.0.2	*Ddx3a*	XM_008279959.1
			Ddx3b	XM_008292391.1
Poecilia reticulate	guppy	Guppy_female_1.0+MT	*Ddx3a*	XM_008437108.1

			Ddx3b	XM_008426133.1
Oreochromis niloticus	Nile tilapia	Orenil1.1	*Ddx3a*	XM_005475255.1
			Ddx3b	NT_167568.1
Cynoglossus semilaevis	tongue sole	Cse_v1.0	*Ddx3a*	XM_008327813.1
			Ddx3b	XM_008324681.1
Takifugu rubripes	torafugu	FUGU5	*Ddx3a*	XM_003961572.1
			Ddx3b	XM_003966411.1
Oryzias latipes	Japanese medaka	ASM31367v1	*Ddx3a*	XM_004081602.1
			Ddx3b	XM_004085517.1
Paralichthys olivaceus	Japanese flounder	unpublished	*Ddx3a*	KP205082
			Ddx3b	KP205083
Poecilia formosa	Amazon molly	Poecilia_formosa-5.1.2	*Ddx3a*	XM_007571428.1
			Ddx3b	XM_007572225.1
Xiphophorus maculatus	southern platyfish	Xiphophorus_	*Ddx3a*	XM_005803788.1
		maculatus-4.4.2	*Ddx3b*	XM_005813094.1
Maylandia zebra	zebra mbuna	MetZeb1.1	*Ddx3a*	XM_004566279.1
			Ddx3b	XM_004573843.1
Tetraodon nigroviridis	tetraodon	TETRAODON 8.0	*Ddx3a*	ENSTNIG00000011569
			Ddx3b	ENSTNIG00000014514
Gasterosteus aculeatus	stickleback	BROAD S1	*Ddx3a*	ENSGACG00000008206
			Ddx3b	ENSGACG00000014407
