# Supplementary material for: Comparative Evolution of Duplicated Ddx3 Genes in Teleosts: Insights from Japanese Flounder, Paralichthys olivaceus
Source: G3 (Bethesda). 2015 Jun 24;5(8):1765–73. doi: 10.1534/g3.115.018911 (PMC4528332; doi:10.1534/g3.115.018911)
Supplement: Supporting Information [file supp_g3.115.018911_TableS2.pdf]

Table S2 Sequences of primers used for cloning Japanese flounder *Ddx3* genes.

Primers	Sequence(5’-3’)	Product	usage
ddx3-1_FW	TTTCTGTACCACGAAGGTTACGC	1370 bp	PCR
ddx3-1_RV	CATAGCCGTCATTGCTGTAGAAG		
ddx3-2_FW	ATTGGAACAGGAGCATTTGACC	512 bp	PCR
ddx3-2_RV	GTCAGAGGCTTTGAATGGGGT		
DDX3-1-FW	GATGCTCCAAAAAACGATTCAC	128 bp	RT-qPCR
DDX3-1-RV	ATTCCTCTATCATTGCGAGGG		
DDX3-2-FW	ATGCCATTCCAATCGTCAAG	289 bp	RT-qPCR
DDX3-2-RV	TACACAACACAGGGACGCACT		
