# Supplementary material for: Comparative Evolution of Duplicated Ddx3 Genes in Teleosts: Insights from Japanese Flounder, Paralichthys olivaceus
Source: G3 (Bethesda). 2015 Jun 24;5(8):1765–73. doi: 10.1534/g3.115.018911 (PMC4528332; doi:10.1534/g3.115.018911)
Supplement: Supporting Information [file supp_g3.115.018911_TableS3.pdf]

Table S3 Results of site model analyses on teleost *Ddx3* Bayesian gene trees.

Tree	Model	ln*L*	*κ*	Parameters[a]			Null	LRT	df	*P*
				ω_0/p	ω_1/q	ω_2/ω_p				
Ddx3a	M0	-12032.610	1.827	0.077			n/a			
	M1a	-11821.569	1.941	0.042(89.94%)	1.000(10.06%)		n/a			
	M2a	-11821.569	1.941	0.042(89.94%)	1.000(10.06%)	87.302(0)	M1a	0	2	**1.000**
	M3	-11730.857	1.829	0.013(74.72%)	0.224(20.60%)	0.750(4.68%)	M0	603.506	4	**0**
	M7	-11734.989	1.823	0.198	1.828		n/a			
	M8a	-11739.484	1.849	0.223	2.257	1.000(3.71%)	n/a			
	M8	-11731.240	1.825	0.207	2.044	2.600(0.35%)	M7	7.498	2	**0.024**
							M8a	16.488	1	**0**
Ddx3	M0	-10420.556	1.782	0.057			n/a			
	M1a	-10373.0	1.857	0.046(95.77%)	1.000(4.23%)		n/a			

	22								
M2a	-10373.022	1.857	0.046(95.77%)	1.000(4.23%)	48.098 (0)	M1a	0	2	1.000
M3	-10288.100	1.775	0(52.95%)	0.084(37.85%)	0.324(9.20%)	M0	264.912	4	0
M7	-10288.873	1.776	0.286	4. 080		n/a			
M8a	-10287.692	1.774	0.230	2. 738	1.000(0.65%)	n/a			
M8	-10286.625	1.782	0.306	4.687	1.449(0.41%)	M7	4.496	2	0.106
						M8a	2.134	1	0.144

Abbreviations: ln*L*, *ln* likelihood; ***κ***, transition/transversion ratio; LRT, likelihood ratio test statistic; df, degrees of freedom; ***P***, P-value; n/a, not applicable.

[a] : ω values of each site class are shown for models M0-M3 (ω_0-ω_2) with the proportion of each site class in parentheses. For M7-M8, the shape parameters, p and q, which describe the beta distribution are listed. In addition, the ω value for the positively selected site class (ω_p, with the proportion of sites in parentheses) is shown for M8a (where ω_p is constrained to equal one) and M8.
