# Supplementary material for: Comparative Evolution of Duplicated Ddx3 Genes in Teleosts: Insights from Japanese Flounder, Paralichthys olivaceus
Source: G3 (Bethesda). 2015 Jun 24;5(8):1765–73. doi: 10.1534/g3.115.018911 (PMC4528332; doi:10.1534/g3.115.018911)
Supplement: Supporting Information [file supp_g3.115.018911_TableS4.pdf]

Table S4 Sites selected in *Ddx3a* by BEB in Bayesian tree.

Model	codon site	BEB		
		Pr(ω>1)	post mean	+/-
M8	43	0.513	1.070	0.459
	89	0.583	1.132	0.451
	131	0.578	1.135	0.443
	151	0.577	1.140	0.436
	176	0.860	1.392	0.275
	199	0.571	1.136	0.435
	219	0.562	1.124	0.441
	429	0.962*	1.473	0.147
	607	0.539	1.093	0.458
	609	0.560	1.089	0.483
	616	0.980*	1.486	0.109
	620	0.763	1.306	0.358

*: posterior probability > 0.95
