# Supplementary material for: Comparative Evolution of Duplicated Ddx3 Genes in Teleosts: Insights from Japanese Flounder, Paralichthys olivaceus
Source: G3 (Bethesda). 2015 Jun 24;5(8):1765–73. doi: 10.1534/g3.115.018911 (PMC4528332; doi:10.1534/g3.115.018911)
Supplement: Supporting Information [file supp_g3.115.018911_TableS5.pdf]

Table S5 Results of site model analyses on teleost *Ddx3* ML gene trees.

Tree	Model	ln*L*	κ	Parameters[a]			Null	LRT	df	*P*
				ω_0/p	ω_1/q	ω_2/ω_p				
Ddx3a	M0	-12069.904	1.870	0.079			n/a			
	M1a	-11828.656	1.967	0.042(90.37%)	1.000(9.63%)		n/a			
	M2a	-11828.656	1.967	0.042(90.37%)	1.000(9.63%)	37.503(0)	M1a	0	2	1.000
	M3	-11746.341	1.872	0.013(74.49%)	0.217(20.62%)	0.813(4.89%)	M0	647.126	4	0
	M7	-11753.109	1.865	0.190	1.672		n/a			
	M8a	-11753.656	1.891	0.224	2.438	1.000(4.47%)	n/a			
	M8	-11745.686	1.873	0.242	3.063	1.000(2.94%)	M7	14.846	2	0
							M8a	15.940	1	0
Ddx3b	M0	-10427.457	1.799	0.057			n/a			
	M1a	-10379.321	1.873	0.046(95.89%)	1.000(4.11%)		n/a			
	M2a	-10379.321	1.873	0.046(95.89%)	1.000(4.11%)	50.201 (0)	M1a	0	2	1.000
	M3	-10291.089	1.797	0.010(71.32%)	0.178(28.17%)	1.431(0.51%)	M0	272.736	4	0
	M7	-10293.577	1.793	0.285	4. 030		n/a			
	M8a	-10292.146	1.791	0.230	2. 714	1.000(0.67%)	n/a			
	M8	-	1.800	0.306	4.651	1.494(0.42%)	M7	4.892	2	0.087

10291.131				
	M8a	2.03	1	**0.154**

Abbreviations: **ln*L***, *ln* likelihood; ***κ***, transition/transversion ratio; **LRT**, likelihood ratio test statistic; **df**, degrees of freedom; ***P***, P-value; **n/a**, not applicable.

[a] : ω values of each site class are shown for models M0-M3 (ω_0-ω_2) with the proportion of each site class in parentheses. For M7-M8, the shape parameters, p and q, which describe the beta distribution are listed. In addition, the ω value for the positively selected site class (ω_p, with the proportion of sites in parentheses) is shown for M8a (where ω_p is constrained to equal one) and M8.
