# Supplementary material for: Comparative Evolution of Duplicated Ddx3 Genes in Teleosts: Insights from Japanese Flounder, Paralichthys olivaceus
Source: G3 (Bethesda). 2015 Jun 24;5(8):1765–73. doi: 10.1534/g3.115.018911 (PMC4528332; doi:10.1534/g3.115.018911)
Supplement: Supporting Information [file supp_g3.115.018911_TableS6.pdf]

Table S6 Sites selected in *Ddx3a* by BEB in ML tree.

Model	codon site	BEB		
		Pr(ω>1)	post mean	+/-
M8	9	0.585	1.127	0.460
	43	0.604	1.157	0.438
	89	0.581	1.129	0.453
	111	0.651	1.198	0.426
	123	0.842	1.368	0.314
	176	0.850	1.383	0.285
	199	0.765	1.309	0.353
	219	0.654	1.211	0.409
	429	0.983*	1.488	0.094
	607	0.791	1.329	0.341
	609	0.544	1.071	0.488
	616	0.984*	1.489	0.092
	620	0.837	1.370	0.301

*: posterior probability > 0.95
